# Accumulation of spontaneous γH2AX foci in long-term cultured mesenchymal stromal cells

**DOI:** 10.18632/aging.101142

**Published:** 2016-12-11

**Authors:** Margarita Pustovalova, Anna Grekhova, Tatiana Astrelina, Viktoria Nikitina, Ekaterina Dobrovolskaya, Yulia Suchkova, Irina Kobzeva, Darya Usupzhanova, Natalia Vorobyeva, Aleksandr Samoylov, Andrey Bushmanov, Ivan V. Ozerov, Alex Zhavoronkov, Sergey Leonov, Dmitry Klokov, Andreyan N. Osipov

**Affiliations:** ^1^ State Research Center-Burnasyan Federal Medical Biophysical Center of Federal Medical Biological Agency (SRC-FMBC), Moscow 123098, Russia; ^2^ Insilico Medicine, Inc., Emerging Technology Centers, Johns Hopkins University at Eastern, Baltimore, MD 21218, USA; ^3^ Life Sciences Center, Moscow Institute of Physics and Technology, Dolgoprudny, Moscow Region 141700, Russia; ^4^ Canadian Nuclear Laboratories, Chalk River, ON K0J1P0, Canada

**Keywords:** mesenchymal stromal cells, long-term cultivation, genome instability, DNA double-strand breaks, γH2AX foci, replicative senescence, cellular senescence

## Abstract

Expansion of mesenchymal stromal/stem cells (MSCs) used in clinical practices may be associated with accumulation of genetic instability. Understanding temporal and mechanistic aspects of this process is important for improving stem cell therapy protocols. We used γH2AX foci as a marker of a genetic instability event and quantified it in MSCs that undergone various numbers of passage (3-22). We found that γH2AX foci numbers increased in cells of late passages, with a sharp increase at passage 16-18. By measuring in parallel foci of ATM phosphorylated at Ser-1981 and their co-localization with γH2AX foci, along with differentiating cells into proliferating and resting by using a Ki67 marker, we conclude that the sharp increase in γH2AX foci numbers was ATM-independent and happened predominantly in proliferating cells. At the same time, gradual and moderate increase in γH2AX foci with passage number seen in both resting and proliferating cells may represent a slow, DNA double-strand break related component of the accumulation of genetic instability in MSCs. Our results provide important information on selecting appropriate passage numbers exceeding which would be associated with substantial risks to a patient-recipient, both with respect to therapeutic efficiency and side-effects related to potential neoplastic transformations due to genetic instability acquired by MSCs during expansion.

## INTRODUCTION

Currently, mesenchymal stromal/stem cells (MSCs) derived from various sources (tissues) are often used for cell based therapies to treat a variety of diseases [1]. Such applications typically require large numbers of cells produced by *in vitro* expansion of cells via continuing passaging. However, as the passage number increases, the risk of genetic alterations also increases.

Indeed, high passage numbers in MSCs have been shown to contribute to the formation of chromosomal aberrations [2, 3], the inability of cells to differentiate, and oncogenic transformation [4-6]. It is generally assumed that these effects are associated, through unknown mechanisms, with the process of replicative senescence, or aging, of cells [7]. However, substantial gaps in our knowledge of the genetic instability in long-term cultivated MSCs still exist. Unsolved questions include both the evaluation criteria and mechanisms of genetic instability in MSCs during cultivation, as well as the therapeutic time window, i.e. the critical number of cell passages suitable for clinical use.

Accumulation of DNA damage due to incomplete or inaccurate repair of spontaneous DNA lesions (caused by metabolic free radicals, replication and recombination errors, spontaneous chemical modifications) is the most significant contributor to genetic instability in cells that have not been exposed to external DNA damaging stimuli, such as ionizing radiation, UV, chemicals, etc. [8]. Some authors consider the accumulation of DNA damage in cells as a universal cause of age-dependent changes in cells [9, 10]. Among the variety of spontaneous DNA lesions, most of the interest of researchers has focused on DNA double-strand breaks (DSB). Indeed, DSBs are the most critical DNA alterations that can define the fate of cells and, if repaired incorrectly or inefficiently, can lead to serious cytogenetic abnormalities, cell death, inactivation of tumor suppressor genes or activation of oncogenes [11-14]. Moreover, in recent years, functional state of DNA DSB repair systems, as well as accumulation of DSB, have been linked to the formation of a particular phenotype inherent to aging cells [15].

An indirect method based on immunofluorescence microscopy analysis of proteins involved in DSB repair has recently gained broad use to study quantitative DSB-related changes in living cells. Complex dynamic microstructures formed during DNA DSB repair consisting of thousands of copies of proteins and visualized by immunofluorescence staining appear as bright spots of fluorescence, called DNA repair foci [16, 17]. It is believed that one focus is the repair site of one single or multiple DSBs [18]. Notably, the immuno-fluorescence analysis of phosphorylated at serine 139 core histone H2AX (also known as γH2AX) has been the most widely used marker of DNA DSBs [19, 20]. Functioning as a binding site for the protein MDC1, γH2AX recruits key DNA repair proteins [21] and in such way, forming a vital part of the machinery that ensures genome stability. Members of the superfamily of phosphatidylinositol 3-kinase-related kinases (PIKKs), in particular Serine/Threonine protein kinases ATM (Ataxia telangiectasia mutated), ATR (ATM- and RAD3-related) and DNA-PKcs (DNA-dependent protein kinase catalytic subunit), phosphorylate H2AX in response to DSB acting as primary DSB sensor proteins [22].

The aim of our study was to investigate the pattern of change in the number of γH2AX foci during long-term (up to 22 passage) culturing of MSCs.

To reveal possible mechanisms of change in the number of γH2AX foci, we additionally performed: 1) quantitative analysis of activated (sequentially auto-phosphorylated at Ser1981, Ser367 and Ser1893) ATM foci in response to DSBs [23]; 2) differential quantitative analysis of γH2AX foci in actively proliferating (Ki67(+)) and resting (Ki67(−)) MSCs. The associated with ribosomal RNA transcription RNA [24] Ki67 protein is present in actively proliferating (during G1, S, G2 and M phases of the cell cycle), while being absent in resting (G0 phase) cells [25].

## RESULTS

### Quantitative analysis of the γH2AX and pATM foci

**Figure 1:**
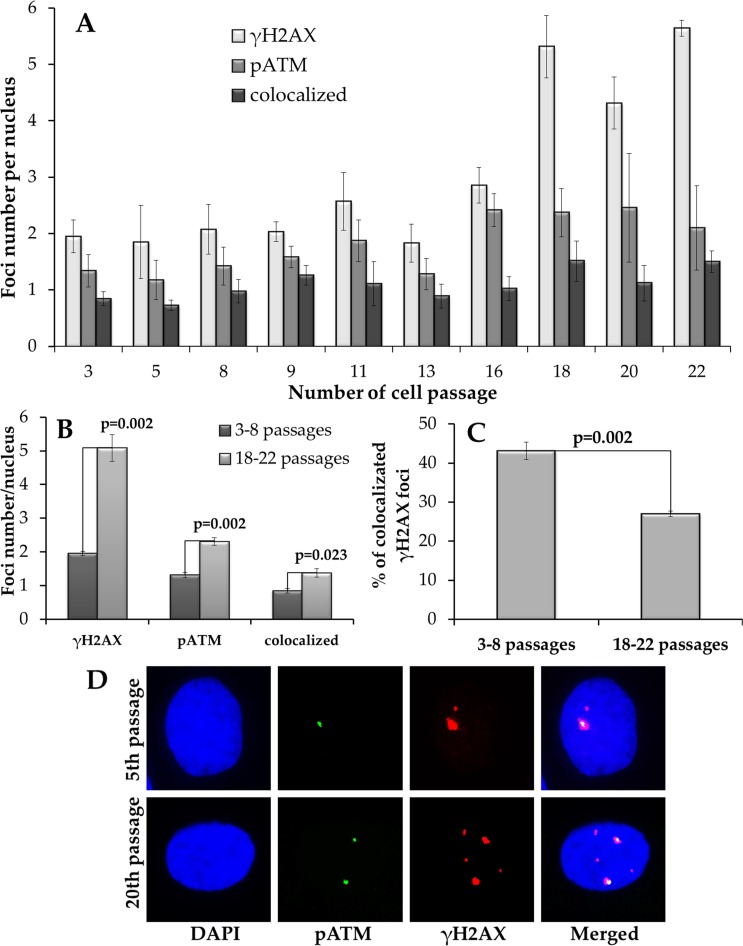
Immunocytochemical analysis of γH2AX and pATM foci (**A**) Changes in γH2AX, pATM foci and their co‐localization depending on the passage number in MSCs. (**B**) Comparative analysis of γH2AX, pATM foci and their co‐localization in early (3‐8) vs. late (18‐22) passages of MSCs. (**C**) Fraction of γH2AX foci that co‐localize with pATM at early (3‐8) vs. late (18‐22) passages of MSCs. (**D**) Representative immunofluorescent microphotographs of MSC showing γH2AX (green), pATM (red) foci and their co‐localization (yellow) at passage 5 and 20. Nuclei were counterstained with DAPI.

Quantification of γH2AX foci in MSCs at different passages is shown in Fig. 1A. It can be seen that between passages 3-16, the number of γH2AX foci did not change (r=0.66; p=0.11), whereas at passages 16-22, the number of the foci doubled. In contrast, phosphorylated ATM (pATM) foci increased gradually with the increase in the passage number (Fig. 1A). The pATM data could be best fit with a linear regression equation y=0.99 + 0.07x (r=0.83; p=0.003), where y is the number of foci per nucleus, X is the passage number. Similar pattern was observed for γH2AX foci co-localized with pATM foci: y=0.72 + 0.03x (r=0.73; p=0.017).

It was interesting to compare directly foci quantities at early (3-8) and late (18-22) passages. As seen in Fig. 1B, both γH2AX and pATM foci significantly increased in late compared with early passage cells. However, whereas the number of γH2AX foci tripled, the increase for pATM foci was only 2-fold. Interestingly, comparing fractions of γH2AX foci co-localized with pATM between the early and late passages showed that the number dropped from 43±2 % foci at passages 3-8 to 27±1 % at passages 18-22. This data indicates that the sharp increase in γH2AX foci at late passages may not be ATM-dependent.

### Differential analysis of γH2AX foci in proliferating and resting cells

**Figure 2:**
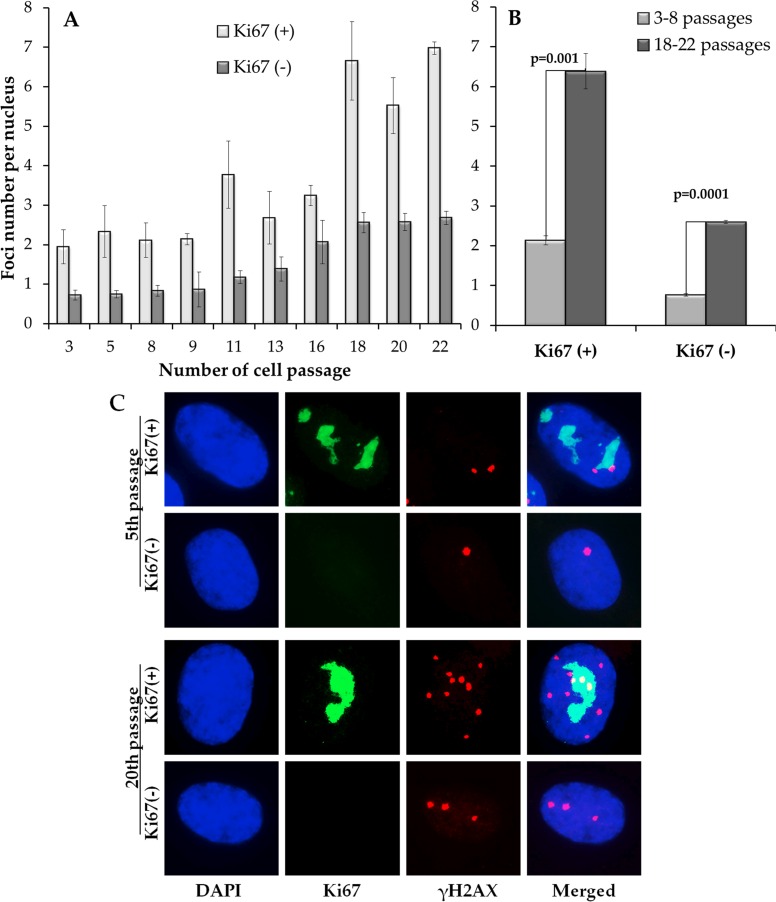
Differential immunocytochemical analysis of γH2AX foci in proliferating (Ki67(+)) and resting (Ki67(−)) cells (**A**) Changes in the γH2AX number in Ki67(+) and Ki67(−) cells on 3-22 passages (**B**) Comparative analysis of γH2AX in Ki67(+) and Ki67(−) cells on early (3-8) vs. late (18-22) passages; (**C**) Representative immunofluorescent microphotographs of MSC showing Ki67 (green), γH2AX (red) foci and their co-localization (yellow) at passage 5 and 20. Nuclei were counterstained with DAPI.

Analysis of γH2AX foci in proliferating Ki67-positive (Ki67(+)) and resting Ki67-negative (Ki67(−)) is presented in Fig. 2. The number of γH2AX foci in proliferating cells was higher than that in resting cells for all examined passage numbers (Fig. 2A). No significant changes in γH2AX foci were found in proliferating cells at passages 3-16, after which the number of foci sharply increased (Fig. 2A). A different pattern was observed in resting cells: the number of γH2AX foci increased more or less gradually with the increase in passage number and, similarly to pATM foci kinetics, was well fit with a linear regression y=0.02 + 0.12x (r=0.96; p=0.00001), where y is the number of γH2AX foci and x is the passage number. Moreover, a statistically significant correlation was found between the number of γH2AX foci in resting cells and the number of pATM foci in all cells (r=0.87; p=0.001).

When early (3-8) and late (16-22) passages were compared directly, the number of γH2AX foci was higher in both proliferating and resting MSC (Fig. 2B). However, absolute numbers of γH2AX foci were different in proliferating vs. resting cells, 4.3 vs. 1.8 foci/nucleus, respectively. These results suggest either a higher rate of DNA DSB induction or a broader spectrum of mechanisms leading to DNA DSBs in proliferating vs. resting cells. However, diminishing DNA DSB repair mechanisms as a source of such difference between proliferating vs. resting cells cannot be ruled out as well.

## DISCUSSION

In this study we showed that long-term culture of MSCs leads to accumulation of γH2AX foci. Late passage cells were characterized by a ∼3-fold increased number of foci compared with early passage cells. Apparently, two parallel processes are involved in the observed accumulation of γH2AX foci that are essentially distinct:

1. Gradual ATM-dependent accumulation of γH2AX foci in long-term cultured MSCs.
2. Step-wise ATM-independent increase of γH2AX foci numbers between passages 16 and 18.

For the first process, the ATM kinase, unlike the ATR kinase that phosphorylates H2AX upon formation of large stretches of single-stranded DNA at collapsed replication forks and nucleotide excision repair sites [26, 27], phosphorylates histone H2AX in response to single or clustered DSBs [28, 29]. Thus, accumulation of γH2AX co-localized with pATM suggests that these foci represent DNA DSBs in long-cultured MSCs. However, these co-localized foci had a minor contribution to an overall passage dependent increase in γH2AX foci (Fig. 1B). Accumulation of DSBs at telomeres may contribute to this process, since it was shown that repair efficiency of DNS DSBs at telomeres is low and may lead to accumulation to γH2AX foci at telomere repeats [30, 31]. This process is related to cellular aging and the accumulation of γH2AX foci in senescent cells [31, 32]. This is consistent with our results showing the accumulation of γH2AX foci in Ki67-negative cells, which may also represent senescent cells. Since passage related γH2AX foci in MSCs co-localized poorly with pATM foci (Fig. 1C), consistent with the results of Pospelova et al. [33], it is likely that they represent non-DNA damage related foci. Such foci have been shown to be associated with mTOR signaling pathway in senescent cells only and could be inhibited by rapamycin [33, 34].

With regards to the second process, it appears to take place exclusively in proliferating cells and to be ATM-independent. This suggests an age-related increase in the rate of errors during DNA replication. For example, DNA DSBs can be formed at collapsed replication forks of late-passage cells due to increased oxidative damage associated with age-dependent dysfunction of mitochondria [35]. Phosphorylation of H2AX by a DNA-PKcs/CHK2 pathway could also contribute to this age-associated process [36].

Another explanation of the increased rates of γH2AX foci in late-passage MSCs could be the inefficient γH2AX de-phosphorylation processes, also in turn related to cellular aging [37, 38]. Moreover, replicative senescence related changes in chromatin remodeling process may also represent the source of additional γH2AX foci in late-passage MSCs [38]. However, this hypothesis is inconsistent with the results of a recent study in which another marker of DNA DSBs, 53BP1 that is not relevant to H2AX, was used [39]. It is not unlikely that diminished rates of ATM-dependent phosphorylation of histone H2AX may indicate an onset of the senescence phenotype. It was shown that the concentration and the level of ATM phosphorylation, which defines its kinase activity, after exposure to ionizing radiation was lower in old compared to young mice [40]. On the contrary, ATM-mediated DNA damage response was shown to prevent further damage, induce SASP and boost protection against malignant transformation [41]. Lowered ATM activity may contribute to diminished p53-dependent responses to DNA damage, induced or spontaneous, leading to subsequent sharp increases in DNA DSBs in MSCs of high passages forming the senescence phenotype. Lastly, various DNA repair pathways were shown to be affected by age in various mouse and human tissues [42-46]. Such changes in DNA repair efficiencies may also contribute to passage-associated accumulation of γH2AX foci in MSCs found in this study. Lastly, it appears that apoptotic cells had no role in the observed accumulation of genetic instability markers as we did not observe an increase in presumptively apoptotic cells containing >25 γH2AX foci per cell, nor did we notice accumulation of nuclei with apoptotic morphology (data not shown).

## CONCLUSIONS

A passage dependent accumulation of γH2AX foci in MSCs, with a sharp increase at passages 16-18, was observed in this study, indicative of genomic instability. It appears that the mechanisms of this increased rates of γH2AX foci include both a ATM-dependent, most likely representing physical DNA DSBs irrespective of cell proliferation status, slow component and a ATM-independent fast component that gets abruptly activated at passage 16-18 in proliferating cells. While precise mechanisms of the two identified components are not clear and represent obvious interest for future studies, our results provide important information with respect to the clinical applications of stem cell therapy. Indeed, understanding how passage number affects genomic instability in MSCs would allow optimizing clinical protocols for *in vitro* expansion of the cells to achieve higher therapeutic outcomes.

## METHODS

### Culture and immunophenotypic characterization of MSCs

MSCs were obtained from mucosa of a 40-year old healthy male donor. Cells were cultured in low glucose DMEM (StemCell, USA) supplemented with L-glutamin, penicillin/streptomycin and 20% fetal bovine serum (StemCell, USA) at a concentration of 0.3 × 10^6^ per flask with filter ventilated caps (25 cm^2^) in a humidified atmosphere of 5% CO_2_ and 37°C. MSCs were subcultured every 7 days up to passage 22.

For immunophenotypic characterization, cells were stained with the panels of antibodies against the following surface markers: CD3, CD13, CD14, CD19, CD25, CD29, CD31, CD34, CD38, CD44, CD45, CD69, CD73, CD90, CD105, CD106, CD166 and HLA-DR (Becton Dickinson, USA). The expression of the surface markers was then analyzed using a BD FACS Canto II (Becton Dickinson Bioscience, USA) flow cytometer. The resulting expression profiles revealed high expression levels (>60% positive cells) for CD90, CD105, CD166, CD44, CD73, medium levels (30-60%) for CD13, CD29 and CD69, and very low levels (<5%) for CD45, CD34, CD133, CD3, CD19, CD25, CD38, CD45, CD106, CD31 markers. This immunophenotype was consistent with the reported immunophenotype for MSCs [47] and did not change in the course of the experiment.

### Immunofluorescence microscopy

MSCs at passages 3, 5, 8, 9, 11, 13, 16, 18, 20 and 22 were detached with 0.25% Trypsin/EDTA (StemCell Technology, USA), washed, resuspended and seeded at the density of 5 × 10^3^ cells/cm^2^ in 500 μL of culture medium onto coverslips (SPL Lifesciences, South Korea) placed inside 35 mm Petri dishes (Corning, USA). To improve adhesion of cells additional volume of culture medium (1, 5 mL) was added into Petri dishes 15 minutes after seeding. Cells seeded on coverslips were incubated at 37°C and 5% CO_2_ for at 48 h prior to fixation.

Cells were fixed on coverslips in 4% paraformaldehyde in PBS (pH 7.4) for 15 min at room temperature followed by two rinses in PBS and permeabilization for 40 min with 0.3% Triton-X100 (in PBS, pH 7.4) supplemented with 2% bovine serum albumin (BSA) to block non-specific antibody binding. Cells were then incubated for 1 hour at room temperature with primary rabbit monoclonal antibody against γH2AX (dilution 1:200, clone EP854(2)Y, Merck-Millipore, USA) and primary mouse monoclonal antibody against phosphorylated ATM protein (dilution 1:200, clone 10H11.E12, Merck-Millipore, USA) or primary mouse monoclonal antibody against Ki67 protein (dilution 1:400, clone Ki-S5, Merck-Millipore, USA) which were diluted in PBS with 1% BSA. Following several rinses with PBS, cells were incubated for 1 hour at room temperature with secondary antibodies IgG (H+L) goat anti-mouse (Alexa Fluor 488 conjugated, dilution 1:600; Merck-Millipore, USA) and goat anti-rabbit (rhodamine conjugated, dilution 1:400; Merck-Millipore, USA) diluted in PBS (pH 7.4) with 1% BSA. Coverslips were then rinsed several times with PBS and mounted on microscope slides with ProLong Gold medium (Life Technologies, USA) with DAPI for DNA counter-staining. Cells were viewed and imaged using Nikon Eclipse Ni-U microscope (Nikon, Japan) equipped with a high definition camera ProgRes MFcool (Jenoptik AG, Germany). Filter sets used were UV-2E/C (340–380 nm excitation and 435–485 nm emission), B-2E/C (465–495 nm excitation and 515–555 nm emission) and Y-2E/C (540–580 nm excitation and 600–660 nm emission). At least 200 cells per data point were imaged. Foci were counted by manual scoring.

### Statistical analyses

Statistical and mathematical analyses of the data were conducted using the Statistica 8.0 software (StatSoft). Data points in Figures are mean values obtained from three independent experiments; error bars are standard errors. Statistical significance was tested using the Student t-test at p < 0.05.
